# What is the level of evidence of what you are reading?

**DOI:** 10.1590/2176-9451.20.4.022-025.ebo

**Published:** 2015

**Authors:** Renato Parsekian Martins, Peter H. Buschang

**Affiliations:** 1 Adjunct professor, Universidade Estadual Paulista (UNESP), School of Dentistry, Program of Orthodontics, Araraquara, São Paulo, Brazil; 2 Full professor, Regent's professor and Director of Orthodontic Research, Texas A & M University, Baylor College of Dentistry, Dallas, Texas, USA

Clinicians are typically not trained to evaluate scientific papers. As such, they often struggle trying to determine the level of evidence provided in the papers they read. Lack of training leads the reader to the fallacy of *argumentum ad verecundiam*when evaluating an article based on the authors' last names or the journal's title. This is a problem, given that the scientific method does not rank authority as a high level of evidence. 

**Figure 1.**
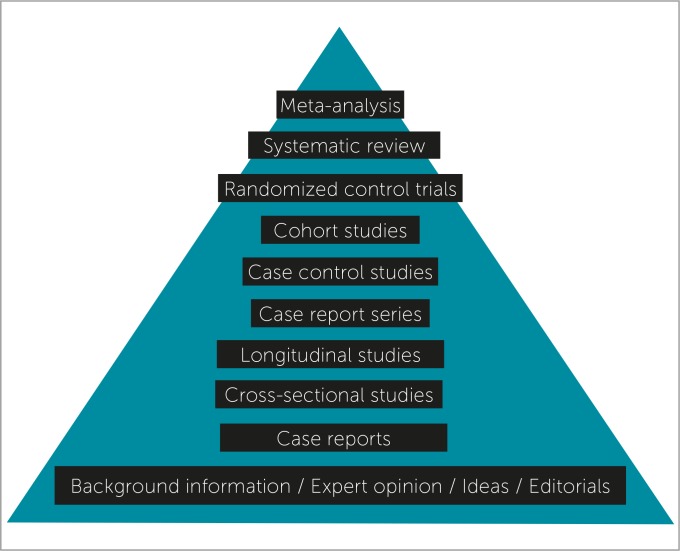
The evidence pyramid.

Today, in the era of evidence-based Dentistry, it is more important than ever to make scientific evidence-based decisions, so as to provide our patients with the best available treatment and enhance treatment efficiency. Thus, in order to do so, clinicians must be able to properly evaluate the literature and be able to identify high-quality evidence. Even though we can organize the level of evidence, based on the type of study (Fig 1), it is imperative that we look upon it with critical eyes and attentive mind, particularly when new or controversial evidence is being presented. 

**Figure 2.**
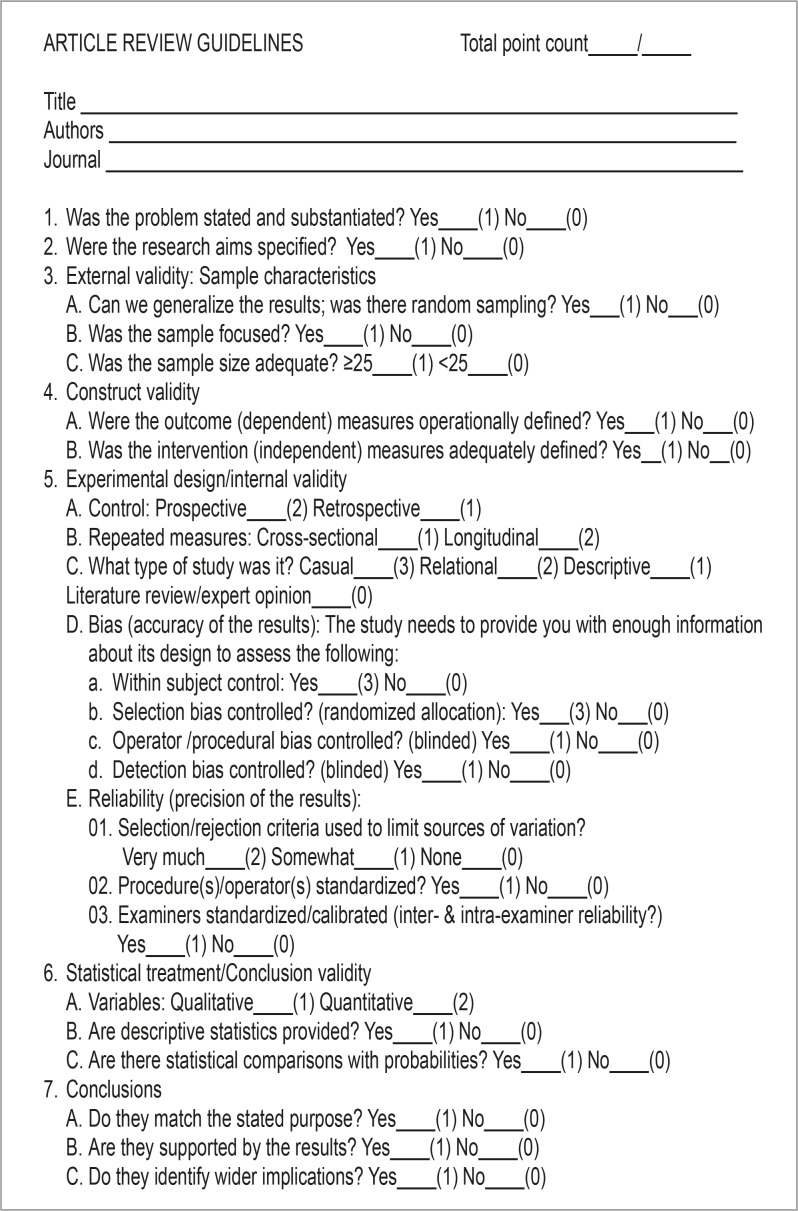
Article review checklist.

A checklist (Fig 2) has been developed by one of the authors to help clinicians quantitatively assess almost any research paper. This checklist was primarily developed for clinical studies, although many of the items also apply to other types of studies. It can be used by reviewers as well as readers to systematically compare studies. Below, we will discuss each one of the checklist items.

The first and second items focus on the purpose of the paper and why a particular investigation should be conducted. Scientific research is always performed with the purpose of answering a question or a set of questions, and it is the author's obligation to make it clear in the introduction what question is supposed to be answered. In a well written introduction, the author correctly states the problem and, supported by existing evidence, concludes that this problem should be solved. If that is stated and substantiated, the paper wins one point. If the aims are clearly specified, the paper wins another point.

The third item evaluates external validity, which pertains to the ability of results to be extrapolated to other people, places or situations. In order to ensure external validity, a random sample from a larger population is needed; should this not be done on a particular paper, multiple studies must be performed in different settings and with different samples in order to ensure it. Another point is granted if the sample is focused. Ideally, the authors should use specific criteria to limit variation and thereby increase the study reliability.

**Figure 3.**
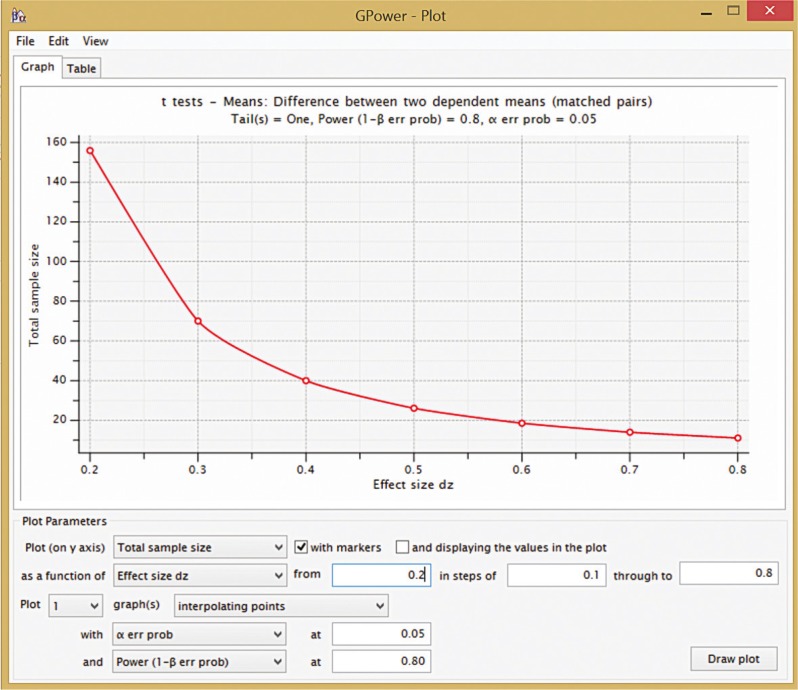
Graph plotted on the software GPower 3.1 showing the sample size needed to detect small to large effect sizes, with probability values of type I error of 0.05 and type II error of 0.20. Small, medium and large effects are usually considered to be 0.2, 0.5, and 0.8, respectively.

Another aspect that has an effect on external validity is sample size. In a well-planned research, sample size should always be calculated a priori (before the research begins), based on the magnitude of expected effects.^1^^,^ 2 ^,^ 3 Research quality is directly related to sample size because the latter partially determines the probability of committing a false-negative statistical error (i.e. saying there is no difference when, in fact, there is). Moreover, a smaller-than-ideal sample might be expected to provide unreliable results, whereas a larger-than-needed sample will spend unnecessary time and money, in addition to the needless exposure of more people. Importantly, a larger-than-needed sample size leads to significant differences that are not clinically meaningful. In order to understand the relationship between effect and sample size, a graph can be plotted with values of sample size needed to detect effect sizes (Fig 3). Usually, a very small sample (< 25) would not be enough to safely detect or rule out differences of a medium effect size (0.5), even on a split-mouth study in which variation is minor. That is why small sample sizes win zero points.

The fourth item pertains to construct validity, which ensures that the variables being measured really reflect what the author wants to measure. For example, assessing changes of the subspinale cephalometric landmark (point A) may not be the best way to access restriction of sagittal maxillary growth, since incisor movement influences the position of this landmark. Both the dependent (the variable that is being tested or measured) independent (the variable that will or will not cause changes in the dependent variable) variables should be evaluated to ensure that they are valid and adequately determined, so as to allow the study to be replicated.

In item five, experimental design and internal validity are evaluated. A prospective control group, collected at the same time as the experimental group, provides a better comparison than a retrospective control group collected fifty years ago. This is because prospective control groups are exposed to the same environmental circumstances as the experimental group. Control groups of a different era, location or culture might respond differently to environment and treatment.

Another item evaluated is the collected data. Repeated measures collected from the same subject over time (longitudinally) may allow cause and effect relationships to be established, something that would not be possible when data are collected from several subjects at only one point in time (cross-sectionally). Causal studies, which are designed to determine when one variable causes or affects an outcome, provide stronger designs than relational studies which are designed to detect relationships established between two or more variables. Observational studies (designed to describe a situation that already exists) provide even weaker designs, followed by expert opinion which is the weakest form of scientific evidence. 

For results to be accurate, known and unknown biases should be controlled. Within subject control, which occurs in split-mouth studies or crossover studies, is probably the best way to control intersubject variability because each person is his/her own control. Even with these designs, random allocation is desirable, with one side of the mouth being randomly assigned as experimental. When within-subject control is not possible, randomization is necessary to ensure internal validity. This is because selection biases of the sample can also be unconsciously inserted in a research. If allocation of a patient who will receive treatment A or B is not random, the researcher in charge of selection could unconsciously create different experimental groups (e.g. if allocation is determined based on facial attributes when comparing extraction *versus* non-extraction treatment, the results might identify differences that are not caused by treatment itself).

Another potential source of bias that should be evaluated is operator and detection bias (which can occur unconsciously). Blinding the operator and/or the statistician is generally done to control these sources of bias. Ideally, patients should also be blinded, otherwise they could influence the results, even on an unconscious level. 

The checklist finally grades some aspects that can improve reliability of results. One of them is the criteria used to select or exclude subjects for the study, in order to keep sources of variation from confounding the results, (i.e. a subject who takes drugs that influence bone remodeling might respond differently to treatment when accessing space closure).

Reliability can also be improved by controlling the procedures being used, which occurs when treatment protocol is well established. This is especially important when the research has multiple operators or is multicenter. For example, if both an experienced orthodontist and a student provide treatment, operator bias could be a problem. In that same context, examiners who are gathering data should be calibrated and trained. Imagine what would happen in a research project that used cephalometric landmarks which are not reliable. That is the reason why errors of measurement should always be accessed in order to assure reliable results.

The authors of good quality papers will normally assess the errors of their measurements by analyzing systematic and random errors.^4^ Systematic errors pertain to consistent differences among measurements, which is a potential problem when different people perform measurements and when the same examiner performs measurements systematically off the true measure that is actually being taken. Random errors pertain to unpredictable errors that occur whenever measurements are taken. This is important because systematic errors will bring biases to measurement, making them invalid, and random errors will increase the size of standard deviation.

Item six grades the analysis of data collected. Parametric tests, which analyze normally distributed quantitative data, are normally more accurate and precise than non-parametric tests. As such, quantitative data win more points, as long as they are normally distributed and a parametric test is applied. If quantitative data are non-normal and non-parametric tests are used, that point is not granted. Descriptive statistics should always be provided in order to quantify existing differences and variation (standard deviation, standard errors, confidence intervals and means). Finally, points are given when the probabilities associated with statistical comparisons are provided. Probabilities of false-positive error (identifying a difference when there is not one) should be considered when differences are detected; while probabilities of false-negative error (not detecting a difference when there is one) should be considered when no differences among groups are found.

The conclusion of a paper should always match the questions proposed (items 1 and 2). Unfortunately, it is not unusual for some papers to "fish" for results, or find statistical differences (by chance) among variables that were not proposed to be measured initially. Thus, whenever a large number of comparisons have been made, they need to be corrected by resampling methods or Bonferroni correction.^6^ Therefore, papers matching purposes and conclusions win points; however, the greatest problem is when a conclusion cannot be supported by the results. The conclusions should not be based on deductions (whose place is in the discussion section). They should be based on the results and identify the wider implications of the findings (such as their clinical importance).

By using this checklist, clinicians who are not comfortable with research design can quantitatively evaluate and analyze the quality of the articles they are reading. Hopefully, it will also serve as guideline for reviewers to objectively assess articles for publication.
